# Force sensor reduced measurement error compared with verbal command during sit‐to‐stand assessment of cerebral autoregulation

**DOI:** 10.14814/phy2.15750

**Published:** 2023-06-12

**Authors:** Alicen A. Whitaker, Eric D. Vidoni, Robert N. Montgomery, Kailee Carter, Katelyn Struckle, Sandra A. Billinger

**Affiliations:** ^1^ Department of Physical Therapy, Rehabilitation Science, and Athletic Training University of Kansas Medical Center Kansas City Kansas USA; ^2^ Department of Physical Medicine and Rehabilitation Medical College of Wisconsin Milwaukee Wisconsin USA; ^3^ Cardiovascular Center Medical College of Wisconsin Milwaukee Wisconsin USA; ^4^ University of Kansas Alzheimer's Disease Research Center Fairway Kansas USA; ^5^ Department of Neurology University of Kansas Medical Center Kansas City Kansas USA; ^6^ Department of Biostatistics & Data Science University of Kansas Medical Center Kansas City Kansas USA; ^7^ Department of Physical Medicine and Rehabilitation University of Kansas Medical Center Kansas City Kansas USA; ^8^ Department of Cell Biology and Physiology University of Kansas Medical Center Kansas City Kansas USA

**Keywords:** cerebral hemodynamics, dCA, dynamic cerebral autoregulation, MCAv, middle cerebral artery blood velocity, stroke

## Abstract

Current methods estimate the time delay (TD) before the onset of dynamic cerebral autoregulation (dCA) from verbal command to stand. A force sensor used during a sit‐to‐stand dCA measure provides an objective moment an individual stands (arise‐and‐off, AO). We hypothesized that the detection of AO would improve the accuracy of TD compared with estimation. We measured middle cerebral artery blood velocity (MCAv) and mean arterial pressure (MAP) for 60 s sitting followed by 2‐min standing, three times separated by 20 min. TD was calculated as the time from: (1) verbal command and (2) AO, until an increase in cerebrovascular conductance index (CVCi = MCAv/MAP). Sixty‐five participants were enrolled: young adults (*n* = 25), older adults (*n* = 20), and individuals post‐stroke (*n* = 20). The TD calculated from AO ($\bar{x}$ = 2.98 ± 1.64 s) was shorter than TD estimated from verbal command ($\bar{x}$ = 3.35 ± 1.72 s, *η* ^2^ = 0.49, *p* < 0.001), improving measurement error by ~17%. TD measurement error was not related to age or stroke. Therefore, the force sensor provided an objective method to improve the calculation of TD compared with current methods. Our data support using a force sensor during sit‐to‐stand dCA measures in adults across the lifespan and post‐stroke.

## 1 INTRODUCTION

Dynamic cerebral autoregulation (dCA) is the ability of the brain to independently react to increases or decreases in peripheral mean arterial pressure (MAP) and maintain cerebrovascular stability (Aaslid et al., 1989; Diehl et al., 1995; Newell et al., 1994; van Beek et al., 2008). The dCA response can be measured during a sit‐to‐stand procedure, which represents a meaningful, everyday activity and could be an important assessment in older adults and those with chronic health conditions (Labrecque et al., 2017; Sorond et al., 2009; van Beek et al., 2008). During a sit‐to‐stand procedure, MAP rapidly decreases due to gravity and peripheral vasodilation. Therefore, the dCA response must quickly increase the cerebrovascular conductance index (CVCi) of middle cerebral artery blood velocity (MCAv) to maintain homeostasis (Labrecque et al., 2017; Sorond et al., 2009).

One physiological metric of the sit‐to‐stand dCA measurement is the time delay (TD) before the onset of the regulation, which measures how quickly the cerebrovascular system responds to a drop in MAP by increasing CVCi (Labrecque et al., 2017, 2019; Sorond et al., 2009). The TD before the onset of the regulation response typically occurs within ~10 s of standing (Labrecque et al., 2017, 2019; Serrador et al., 2005; Sorond et al., 2009) but can occur on average ~1.5 s after standing in healthy young men (Labrecque et al., 2017). Therefore, the TD before the onset of the regulation response is an important temporal measure of cerebrovascular function and requires accuracy and precision.

The current method to identify the cerebrovascular TD response has been calculated from the time in which individuals are verbally told to stand up from a seated position. To improve standardization in the assessment of dCA, previous studies have implemented a goniometer to detect the angle and speed of the sit‐to‐stand (Barnes et al., 2018, 2021; Barnes, Ball, Haunton, et al., 2017; Barnes, Ball, Panerai, et al., 2017; Batterham et al., 2020; Klein et al., 2020; Panerai et al., 2021). Our laboratory has focused on older adults and those with physical limitations and wanted to further explore an objective measure of stance time. Our initial work in this line of scientific inquiry described the construction of the force sensor, the method used, and established a “proof of principle” that the force sensor could identify the precise moment of stance and be successfully integrated into our cerebrovascular physiology methods to provide a more standardized approach of the TD response of dCA measures (Whitaker et al., 2022). Furthermore, the code developed for use with the sensor identified the exact moment of transition between sitting and standing (arise‐and‐off, AO; Whitaker et al., 2022). For example, we showed that one individual post‐stroke stood prior to the completion of the verbal command, which potentially introduces measurement error into the dCA response (Whitaker et al., 2022). While our initial publication described the development of the force sensor and provided preliminary support for its use, the next logical step to advance in this line of research was to rigorously test whether measurement error was systematically reduced with the force sensor compared with verbal command across a group of young adults, older adults, and individuals post‐stroke.

Therefore, the objective of this study was to determine whether implementing a force sensor to detect the exact moment of AO would significantly improve the accuracy of the calculation of the TD response compared with the current TD method from the verbal command to stand. We hypothesized that using a force sensor to measure AO to calculate the TD response during a sit‐to‐stand measure of dCA would be significantly more accurate than the estimated TD response from the verbal command to stand in healthy young adults, older adults, and individuals with stroke. We also hypothesized that the measurement error of the TD response would be significantly associated with the following participant demographics: age, body mass index (BMI), fast stance time, and history of stroke.

## 2 MATERIALS AND METHODS

This study reports on the accuracy of the current method used in an ongoing study (NCT04673994). The inclusion and exclusion criteria of this study have been published previously (Whitaker et al., 2022). Briefly, we enrolled healthy young adults 18–30 years old with low cardiovascular risk (Thompson et al., 2013). Older adults and individuals post‐stroke (6 months—5 years ago) were (1) age 40–80 years old, (2) sedentary (<150 min brisk exercise/week; Thompson et al., 2013), (3) able to answer consenting questions and follow a 2‐step command, (4) able to stand up from a chair without physical assistance, and (5) not diagnosed with another underlying neurological disease. Individuals with chronic stroke were included within this study as they are a clinical population that presents with hemiplegia and a slower sit‐to‐stand response that may be detected by the force sensor.

The Human Subjects Committee within the University of Kansas Medical Center's Institutional Review Board approved the study. Prior to starting the study, all individuals were informed of study procedures, benefits, and risks, and asked to provide voluntary written consent. We collected demographic information following written informed consent.

For all procedures, we maintained a constant temperature (22–24°C) and the room dimly lit. On the day of the visit, participants were asked to take their medications as prescribed, not to have caffeine for at least 8 h (Addicott et al., 2009; Institute of Medicine, 2001; Perod et al., 2000), not to perform vigorous exercise for 24 h (Burma et al., 2020), and to abstain from alcohol for 24 h (Mathew & Wilson, 1986). For premenopausal women, we collected data on Days 1–6 of the menstrual cycle (Billinger et al., 2017; Whitaker et al., 2022). Participants were seated with their feet flat on the ground and an upright trunk posture. To determine whether individuals had the leg strength and balance to perform a sit‐to‐stand independently, a standardized 5× sit‐to‐stand was performed. Participants were asked to stand up and sit down five times as quickly as they could without the use of their arms and the time it took to perform the 5× sit‐to‐stand was measured in seconds (Mong et al., 2010; Ng, 2010; Tiedemann et al., 2008). Equipment was then donned, which included (1) bilateral TCD probes (2‐MHz, Multigon Industries Inc.) to measure MCAv, (2) a finger cuff was placed on the left middle finger (or upper extremity without spasticity for individuals post‐stroke) to measure beat‐to‐beat MAP (Finometer, Finapres Medical Systems), (3) a 5‐lead electrocardiogram (ECG; Cardiocard, Nasiff Associates) to measure heart rate, and (4) a nasal cannula attached to a capnograph (BCI Capnocheck Sleep 9004 Smiths Medical) to measure end‐tidal carbon dioxide (P_ET_CO_2_). As reported in our prior work (Whitaker et al., 2022), participants were instructed to place their hand with the finger plethysmograph flat on their chest at heart level and were fitted with an arm sling to hold the Finometer in place (Labrecque et al., 2017; Lipsitz et al., 2000; Sorond et al., 2009).

Our custom force sensor was then placed underneath the participant at the level of their right ischial tuberosity. For individuals with stroke, the force sensor was placed underneath the nonaffected lower extremity (Brunt et al., 2002; Whitaker et al., 2022). Participants performed seated rest for 60 s. The participant was then given a 3‐s countdown and asked to stand at the 60‐s mark. The participant continued standing for an additional 2 min for hemodynamic stability (Drapeau et al., 2019). All measures were recorded at 500 Hz using a custom written software within MATLab, implementing the Data Acquisition Toolbox (R2019a; The Mathworks Inc.). Participants performed three sit‐to‐stand procedures (T1, T2, and T3) during the study visit, each separated by 20 min.

### 2.1 Data analysis

Offline processing of the collected data was done using custom written software within MATLab.

To analyze, the data were divided by R‐to‐R cardiac interval. For each cardiac cycle, mean finger arterial pressure and MCAv were calculated as the area under the curve, as described in previous work (Billinger et al., 2017; Ward et al., 2018).

The left MCAv signal was used for both healthy young adults and older adults as our prior work showed no significant difference between the right and left MCAv (Billinger et al., 2017). However, the right MCAv signal was used if the left was not obtainable (Billinger et al., 2017). In individuals post‐stroke, the ipsilesional hemisphere's MCAv signal was used. As previously published, AO was identified as the minimum of the second derivative of the recorded force sensor voltage upon standing (Whitaker et al., 2022). The manual identification of TD before the onset of the regulation response was completed by two trained researchers (A.W. and K.C.) and evaluated as the physiological beat after standing where there was a continuous increase in CVCi and is defined as CVCi = MCAv/MAP (Labrecque et al., 2017; Lind‐Holst et al., 2011; Whitaker et al., 2022).

The primary aim was to determine whether the force sensor improved the calculation of the cerebrovascular TD response when using AO compared with the estimated time of stance (from verbal command at 60 s) with a two‐way repeated measures ANOVA with a within‐subjects effect for time (T1, T2, and T3) and type of calculation (AO or Estimated). Across time points (T1, T2, and T3), post hoc Wilcoxon signed rank *t*‐tests were used to differences between AO and Estimated. We performed a mixed model ANOVA to test differences in the AO response using a within‐subjects effect for time (T1, T2, and T3) and between‐subjects effect for group (young adult, older adult, and individuals post‐stroke).

The force sensor measures the true time of stance with AO. To analyze measurement error, we calculated the difference between the AO TD response and the estimated TD response from 60 s, which is the current method used in sit‐to‐stand procedures. The average TD measurement error was then plotted within a histogram graph to show the frequency distribution. To further explore measurement error, we calculated the coefficient of variation (CoV = (standard deviation/mean) × 100). Finally, Spearman correlations were then used to determine whether measurement error was significantly related to age and the 5× sit‐to‐stand. Pearson correlations were used to determine whether measurement error was significantly related to BMI and whether a participant had a history of stroke. Normality was checked using a Shapiro–Wilk test.

## 3 RESULTS

Sixty‐five individuals enrolled into the study. Participant characteristics are shown in Table 1.

**TABLE 1:** Participant characteristics.

	Young adults (*n* = 25)	Older adults (*n* = 20)	Stroke (*n* = 20)	*p*‐Value
Age (years)	25 ± 2	61 ± 13^a^	60 ± 13^a^	<0.001*
Female, *n* (%)	12 (48%)	6 (30%)	6 (30%)	0.38
Body mass index (kg/m^2^)	23.9 ± 3.8	28.1 ± 6.2^a^	30.7 ± 4.9^a^	<0.001*
Race, *n* (%)				0.02*
White/Caucasian	18 (72%)	17 (85%)	15 (75%)	
Black/African American	0	2 (10%)	5 (25%)	
Asian	6 (24%)	1 (5%)	0	
Native American	1 (4%)	0	1 (5%)	
Ethnicity, *n* (%)				
Hispanic/Latino	1 (4%)	0	0	1.00

*Note*: Means ± standard deviations. Two individuals identified as multiple races/ethnicities. Participant demographics were compared between groups using a one‐way ANOVA or Kruskal–Wallis ANOVA, with post hoc *t*‐tests. For categorical variables, a Fisher's exact or chi‐squared test was used to compare differences between groups.

^a^ Significantly different from young adults.

* Significantly different between groups (*p* < 0.05).

Due to noise in MAP and TCD with standing, 59 individuals had complete data sets with a TD response at all three timepoints. All individuals complied with abstaining from caffeine, vigorous exercise, and alcohol, as instructed. Baseline lower extremity function and hemodynamics between groups are shown in Table 2. Individuals post‐stroke had a significantly slower five times sit‐to‐stand compared with older adults (*p* < 0.001) and young healthy adults (*p* < 0.001). Young adults had a significantly higher resting MCAv compared with individuals post‐stroke (*p* < 0.001) and older adults (*p* < 0.001). There was no difference between groups in resting MAP, HR, or P_ET_CO_2_.

**TABLE 2:** Baseline hemodynamics.

	Young adults (*n* = 22)	Older adults (*n* = 19)	Stroke (*n* = 18)	*p*‐Value
5 Time sit‐to‐stand (s)	8.3 ± 2.1	8.9 ± 2.4	22.9 ± 14.1^a^ ^,^ ^b^	<0.001*
MCAv (cm/s)	65.58 ± 10.32	47.70 ± 13.21^a^	42.70 ± 10.33^a^	<0.001*
MAP (mmHg)	74.91 ± 12.61	80.64 ± 9.91	81.32 ± 12.66	0.17
HR (bpm)	71 ± 11	64 ± 9	68 ± 11	0.10
P_ET_CO_2_	37.89 ± 3.58	35.35 ± 3.56	36.56 ± 4.21	0.11

*Note*: Means ± standard deviations. Comparisons between groups used a one‐way ANOVA or Kruskal–Wallis ANOVA, with post hoc *t*‐tests.

Abbreviations: HR, heart rate; MAP, mean arterial pressure; MCAv, middle cerebral artery blood velocity; P_ET_CO_2_, end‐tidal carbon dioxide.

^a^ Significantly different from young adults.

^b^ Significantly different from older adults.

* Significantly different between groups (*p* < 0.05).

### 3.1 Comparing the TD response using AO versus estimating

**FIGURE 1:**
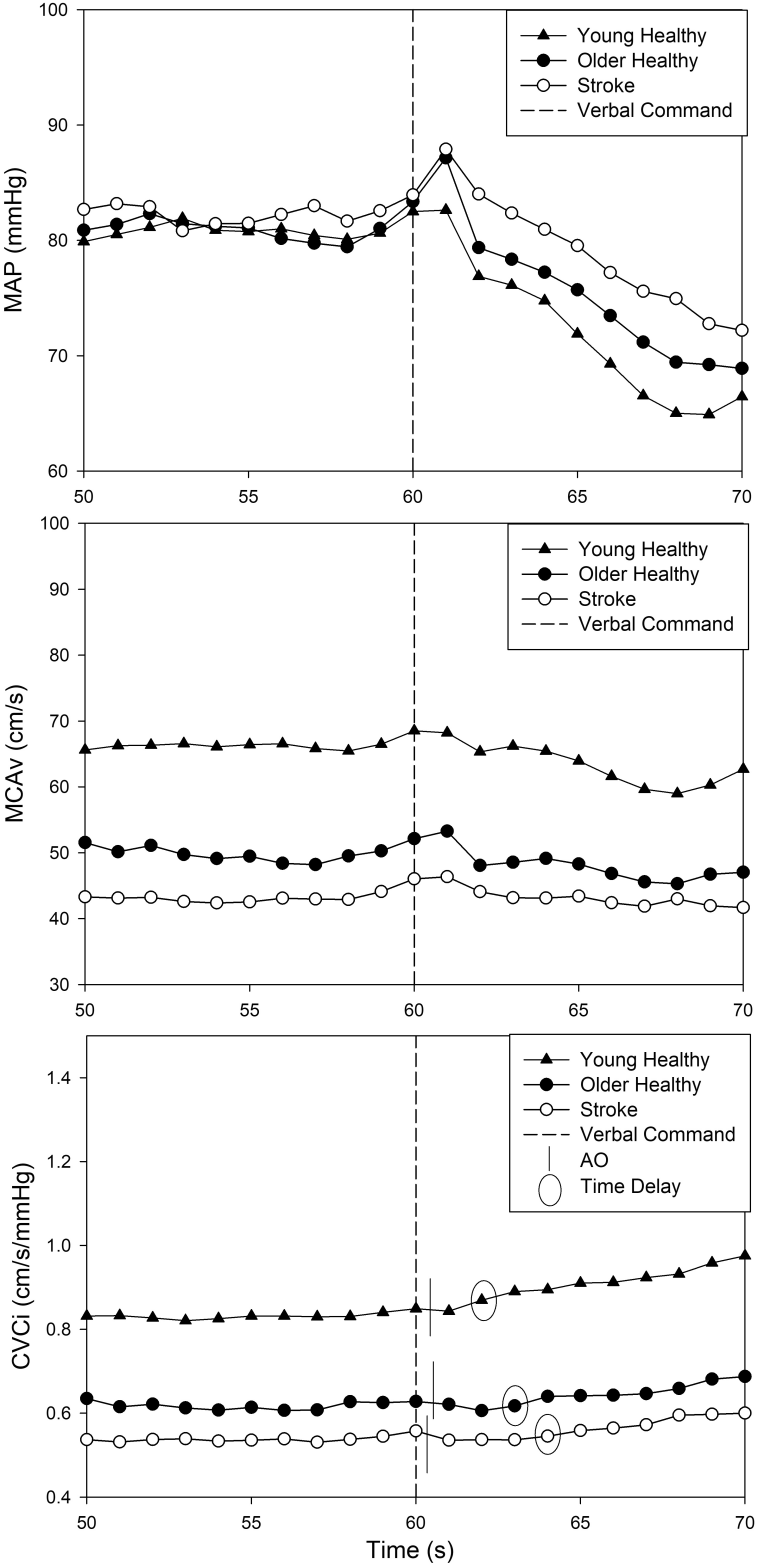
T1 sit‐to‐stand response. Young healthy adults = solid triangles. Older adults = solid circles. Individuals post‐stroke = open circles. AO, arise‐and‐off measured via the force sensor; CVCi, cerebrovascular conductance index (MCAv/MAP); MAP, mean arterial pressure; MCAv, middle cerebral artery blood velocity. Resampled to 1 Hz for group averages.

Our key finding showed a significant difference in TD when using AO compared with estimating from the verbal command to stand at 60 s (*η* ^2^ = 0.49, *p* < 0.001), shown in Figure 1. Across the three trials within the study visit, the TD response was not significantly different (*p* = 0.34). When performing post hoc comparisons, the TD response at each separate time point was significantly shorter when using AO compared with estimating from 60 s (*p* < 0.001). The AO TD response for T1 was $\bar{x}$ = 3.2 ± 2.3 s, T2 was $\bar{x}$ = 2.9 ± 1.9 s, and T3 was $\bar{x}$ = 2.9 ± 2.2 s compared with the estimated TD response of T1 was $\bar{x}$ = 3.7 ± 2.4 s, T2 was $\bar{x}$ = 3.2 ± 2.1 s, and T3 was $\bar{x}$ = 3.2 ± 2.3 s. The data showed no group differences for the measured AO response (*p* = 0.86).

### 3.2 Measurement error compared with verbal command

**FIGURE 2:**
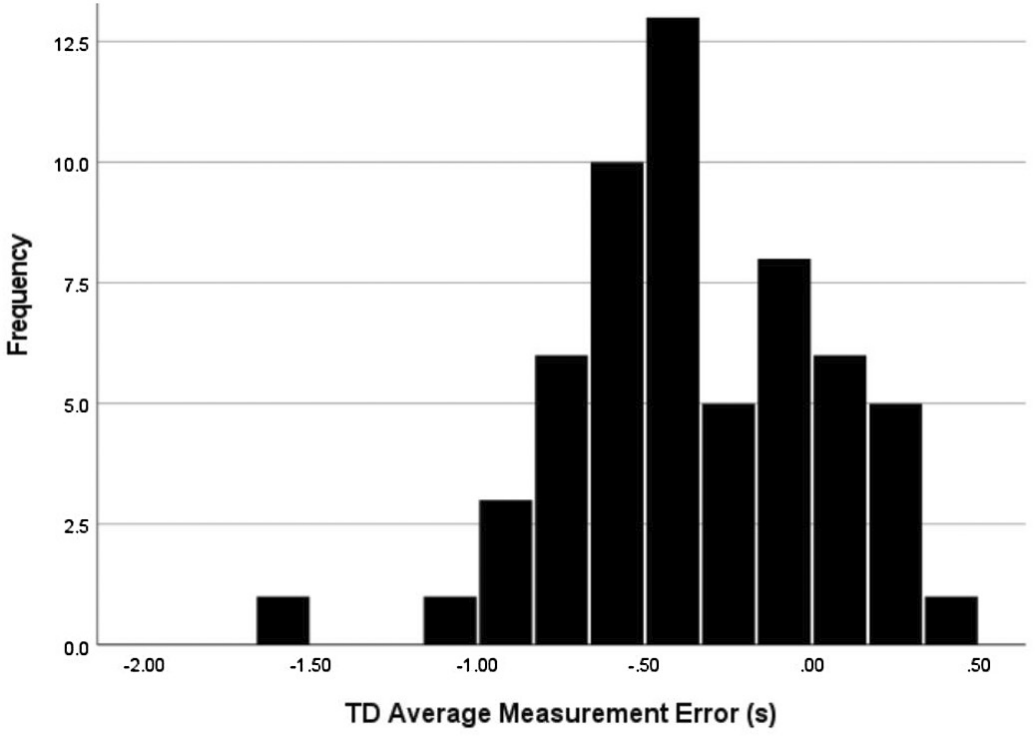
Histogram of the TD average measurement error. TD = time delay.

A histogram in Figure 2 demonstrates the frequency of how predicted values using the current method differ from the true, measured value. The distribution of measurement error is negatively skewed and revealed that stance occurred ~0.5 s after the verbal command. We report the average TD response was 3.0 s with ~17% measurement error when following the current method of estimating the TD response from the verbal command. The maximal error calculated between the AO TD and the TD estimated from 60 s was −2.52 s measured in an older healthy adult.

The average measurement error of the TD response was not significantly related to age (*p* = 0.40), BMI (*p* = 0.73), 5× sit‐to‐stand (*p* = 0.35), or history of stroke (*p* = 0.82).

Data presented in Table 3 show the CoV of AO is small across all groups and suggests low variability across time points and groups.

**TABLE 3:** Coefficient of variation of AO.

	T1			T2			T3		
	AO (s)	TD measurement error (s)	AO CoV	AO (s)	TD measurement error (s)	AO CoV	AO (s)	TD measurement error (s)	AO CoV
Young adults	60.45 ± 0.52	−0.45 ± 0.52	0.85%	60.17 ± 0.49	−0.17 ± 0.49	0.82%	60.29 ± 0.52	−0.29 ± 0.52	0.87%
Older adults	60.54 ± 0.41	−0.54 ± 0.41	0.67%	60.37 ± 0.75	−0.37 ± 0.75	1.25%	60.26 ± 0.47	−0.26 ± 0.47	0.78%
Stroke	60.35 ± 0.65	−0.35 ± 0.65	1.08%	60.41 ± 0.51	−0.41 ± 0.51	0.84%	60.34 ± 0.41	−0.34 ± 0.41	0.68%

*Note*: T1–3 = time points. TD difference = TD AO − TD estimated from 60 s.

Abbreviations: AO, arise‐and‐off (exact moment of stance); CoV, coefficient of variation; TD, time delay.

## 4 DISCUSSION

Using a force sensor to objectively identify AO during a sit‐to‐stand resulted in a more standardized approach during the sit‐to‐stand maneuver when compared to the current method of estimating TD from verbal command to stand. The main findings of this study include (1) the TD response occurred significantly faster when calculated using AO compared with estimating from 60 s, (2) the force sensor reduced measurement error for all individuals *compared with verbal command*, and (3) the AO response showed small variability across time within the same day and across groups consisting of healthy young adults, older adults, and individuals post‐stroke. This study extends our prior work (Whitaker et al., 2022) by showing the force sensor statistically reduced error compared with verbal command during the sit‐to‐stand measure of dCA in not only individuals post‐stroke but also young and older adults.

The reduction in measurement error using an objective identification of stance ensures more accurate reporting of the transient time course changes in MCAv and MAP, which is critical in the field of cerebrovascular physiology and may have implications for dCA. Prior studies have identified two physiological phases that occur in response to the sit‐to‐stand maneuver (Labrecque et al., 2017; Skow et al., 2021). Phase 1 is the initial response and the time point where MCAv responses are “independent of arterial baroreflex input and occur within 1–7 s.” (Deegan et al., 2009; Labrecque et al., 2017; Ogoh et al., 2008; Skow et al., 2021; Sorond et al., 2009; van Beek et al., 2008) Phase 2 continues past Phase 1 and involves the influence of the arterial baroreflex response (Labrecque et al., 2017; Ogoh et al., 2008; Skow et al., 2021). Given the Phase 1 time sensitivity, reducing measurement error in TD would have significant implications in reporting these data. The results reported in this study demonstrate that the force sensor reduced measurement error by ~17%, which would have significant implication for clinical and research applications and may affect dCA results.

Considerable interest exists in understanding cerebrovascular function and brain aging in older adults and clinical populations such as traumatic brain injury, Alzheimer's disease, dementia, and stroke. The sit‐to‐stand maneuver mimics a common and very important daily activity that provides a physiologic challenge. While our intention was to employ a quick sit‐to‐stand response (0–3 s) aligned with the literature (Labrecque et al., 2017), our prior work showed that different stance strategies may be used in older adults and people with stroke. We found some participants, especially those post‐stroke, stood prior to the verbal command (Whitaker et al., 2022), which introduces inherent variability in the sit‐to‐stand maneuver. Furthermore, data in Table 2 reveal a significantly slower 5× sit‐to‐stand time in people with stroke compared with the young and older adults. The implementation of the force sensor to identify the precise moment of stance in clinical populations may have even greater impact on reducing measurement error during a sit‐to‐stand maneuver. Finally, our data support previous findings of low within‐subject variability (Sorond et al., 2009). For all participants regardless of age or stroke, we showed no significant differences in the TD across the three time points, suggesting low variability in this participant sample. While the TD only measures the temporal onset of the regulation response, there are other metrics calculated during the sit‐to‐stand dCA response, such as the rate of regulation, which provides information about the change in CVCi after the TD. Many studies also report dCA in the temporal‐domain alongside measures of dCA in the spectral‐domain (i.e., transfer function analysis) as both techniques provide unique information about cerebrovascular regulation (Klein et al., 2020; Labrecque et al., 2017, 2019).

We acknowledge that the present study did not implement an accelerometer to normalize the dCA response to the speed of the sit‐to‐stand as others have previously done (Barnes et al., 2018, 2021; Barnes, Ball, Haunton, et al., 2017; Batterham et al., 2020; Panerai et al., 2021). Our ongoing work is currently implementing both the force sensor and an accelerometer to identify the precise moment of AO and the speed of stance during the dCA measure. Future studies should also implement the use of a force sensor to determine whether measurement error is reduced and improves the accuracy of the dCA response through transfer function analysis (Burma et al., 2020; Drapeau et al., 2019; Labrecque et al., 2022). Lastly, we report differences in resting MCAv (Table 2) between young adults and older adults and individuals post‐stroke. However, to the extent which the resting MCAv influences AO and the TD presented here is unknown and not within the scope of this project.

In conclusion, our results support the use of a force sensor to reduce TD measurement error during a sit‐to‐stand maneuver. The force sensor improves upon current methods and provides a standardized, objective approach to ensure rigor and reproducibility during a common daily activity, sit‐to‐stand, to assess dCA.

## AUTHOR CONTRIBUTIONS

Alicen A. Whitaker, Eric D. Vidoni, and Sandra A. Billinger conceived and designed research; Alicen A. Whitaker, Kailee Carter, and Katelyn Struckle performed experiments; Alicen A. Whitaker, Kailee Carter, Katelyn Struckle, and Sandra A. Billinger analyzed data; Alicen A. Whitaker, Eric D. Vidoni, Robert N. Montgomery, and Sandra A. Billinger interpreted results of experiments; Alicen A. Whitaker, Eric D. Vidoni, Robert N. Montgomery, Kailee Carter, Katelyn Struckle, and Sandra A. Billinger drafted manuscript; Alicen A. Whitaker, Eric D. Vidoni, Robert N. Montgomery, Kailee Carter, Katelyn Struckle, and Sandra A. Billinger edited and revised manuscript; Alicen A. Whitaker, Eric D. Vidoni, Robert N. Montgomery, Kailee Carter, Katelyn Struckle, and Sandra A. Billinger approved final version of manuscript; Alicen A. Whitaker prepared figures.

## FUNDING INFORMATION

AW was supported by the National Heart, Lung and Blood Institute (T32HL134643), Cardiovascular Center's A.O. Smith Fellowship Scholars Program, Eunice Kennedy Shriver National Institute of Child Health & Human Development of the National Institutes of Health (T32HD057850), and the American Heart Association Predoctoral Fellowship Grant (898190). EV and SB were supported in part by the National Institute on Aging for the KU Alzheimer's Disease Research Center (P30 AG072973). REDCap at the University of Kansas Medical Center is supported by Clinical and Translational Science Awards (CTSA) Award # ULTR000001 from National Center for Research Resources (NCRR). The content is solely the responsibility of the authors and does not necessarily represent the official views of the National Institutes of Health.

## CONFLICT OF INTEREST STATEMENT

The authors declare that the research was conducted in the absence of any commercial or financial relationships that could be construed as a potential conflict of interest.

## ETHICS STATEMENT

The Human subjects Committee and Institutional Review Board at the University of Kansas Medical Center granted ethical approval of this study. All participants were provided verbal and written explanation of the experimental protocol and the associated risks prior to providing written informed consent.

## Data Availability

The datasets analyzed for this study are available upon request to the corresponding author.
